# Diverse electron sources support denitrification under hypoxia in the obligate methanotroph *Methylomicrobium album* strain BG8

**DOI:** 10.3389/fmicb.2015.01072

**Published:** 2015-10-06

**Authors:** K. Dimitri Kits, Dustin J. Campbell, Albert R. Rosana, Lisa Y. Stein

**Affiliations:** Department of Biological Sciences, Faculty of Science, University of AlbertaEdmonton, AB, Canada

**Keywords:** methanotroph, nitrous oxide, denitrification, hypoxia, *Methylomicrobium album* BG8, methane monooxygenase, nitrite reduction

## Abstract

Aerobic methane-oxidizing bacteria (MOB) are a diverse group of microorganisms that are ubiquitous in natural environments. Along with anaerobic MOB and archaea, aerobic methanotrophs are critical for attenuating emission of methane to the atmosphere. Clearly, nitrogen availability in the form of ammonium and nitrite have strong effects on methanotrophic activity and their natural community structures. Previous findings show that nitrite amendment inhibits the activity of some cultivated methanotrophs; however, the physiological pathways that allow some strains to transform nitrite, expression of gene inventories, as well as the electron sources that support this activity remain largely uncharacterized. Here we show that *Methylomicrobium album* strain BG8 utilizes methane, methanol, formaldehyde, formate, ethane, ethanol, and ammonia to support denitrification activity under hypoxia only in the presence of nitrite. We also demonstrate that transcript abundance of putative denitrification genes, *nirS* and one of two *norB* genes, increased in response to nitrite. Furthermore, we found that transcript abundance of *pxmA*, encoding the alpha subunit of a putative copper-containing monooxygenase, increased in response to both nitrite and hypoxia. Our results suggest that expression of denitrification genes, found widely within genomes of aerobic methanotrophs, allow the coupling of substrate oxidation to the reduction of nitrogen oxide terminal electron acceptors under oxygen limitation. The present study expands current knowledge of the metabolic flexibility of methanotrophs by revealing that a diverse array of electron donors support nitrite reduction to nitrous oxide under hypoxia.

## Introduction

Aerobic methane-oxidizing bacteria (MOB) form an important bridge between the global carbon and nitrogen cycles, a relationship impacted by the global use of nitrogenous fertilizers (Bodelier and Steenbergh, 2014). Ammonia (NH_3_) and nitrate (NO_3_^-^) can stimulate the activity of methanotrophs by acting as a nitrogen source for growth and biomass production (Bodelier et al., 2000; Bodelier and Laanbroek, 2004). Further, some methanotrophs such as *Methylomonas denitrificans* utilize NO_3_^-^ as an oxidant for respiration under hypoxia (Kits et al., 2015). Evidently, denitrification in aerobic methanotrophs functions to conserve energy during oxygen (O_2_) limitation (Kits et al., 2015). Alternatively, NH_3_ and nitrite (NO_2_^-^) can act as significant inhibitors of methanotrophic bacteria (King and Schnell, 1994). NH_3_ is a competitive inhibitor of the methane monooxygenase enzyme and NO_2_^-^, produced by methanotrophs that can oxidize NH_3_ to NO_2_^-^, is a toxin with bacteriostatic properties that is known to inhibit the methanotroph formate dehydrogenase enzyme that is essential for the oxidation of formate to carbon dioxide (Dunfield and Knowles, 1995; Cammack et al., 1999; Nyerges et al., 2010).

In spite of the recent discovery that aerobic methanotrophs can denitrify, the energy sources, genetic modules, and environmental factors that govern denitrification in MOB are still poorly understood. *M. denitrificans* FJG1 respires NO_3_^-^ using methane as an electron donor to conserve energy. However, it is not known whether C_1_ energy sources other than CH_4_ (methanol, formaldehyde, and formate) can directly support denitrification. Another possibility, which has not yet been investigated, is that C_2_ compounds (such as ethane and ethanol) and inorganic reduced nitrogen sources (NH_3_) support methanotrophic denitrification. Previous work shows that several obligate methanotrophs, including *Methylomicrobium album* strain BG8, oxidize ethane (C_2_H_6_) and ethanol (C_2_H_6_O) using particulate methane monooxygenase (pMMO) and methanol dehydrogenase (MDH), respectively, even though neither substrate supports growth (Whittenbury et al., 1970; Dalton, 1980; Mountfort, 1990). NH_3_ may be able to support methanotrophic denitrification because many aerobic methanotrophs are capable of oxidizing NH_3_ to NO_2_^-^: a process facilitated by the presence of a copper-containing monooxygenase (CuMMO) enzyme and, in some methanotrophs, a hydroxylamine dehydrogenase homolog (Poret-Peterson et al., 2008). The ability to utilize alternative energy sources to support denitrification would augment the metabolic flexibility of methanotrophs and enable them to sustain respiration in the absence of CH_4_ and/or O_2_.

*Methylomicrobium album* strain BG8 is an aerobic methanotroph that belongs to the phylum Gammaproteo bacteria; the genome lacks a soluble methane monooxygenase but does contain one particulate methane monooxygenase operon (*pmoCAB* – METAL_RS17430, 17425, 17420) and one operon encoding a putative copper monooxygenase (*pxmABC* – METAL_RS06980, 06975, 06970) with no known function. The genome also contains gene modules for import and assimilation of NH_4_^+^ (*amtB –* METAL_RS11045*/gdhB –* METAL_RS11695*/glnA –* METAL_RS11070*/ald –* METAL_RS11565), assimilation of NO_3_^-^ (*nasA –* METAL_RS06040*/nirB –* METAL_RS15330, *nirD* – METAL_RS15325), oxidation of NH_2_OH to NO_2_^-^ (*haoA –* METAL_RS13275), as well as putative denitrification genes – cytochrome *cd*_1_ nitrite reductase (*nirS –* METAL_RS10995), and two copies of cytochrome *c*-dependent nitric oxide reductase (*norB1 –* METAL_RS03925, *norC1* – METAL_RS03930*/norB2 –* METAL_RS13345). The recent release of several genome sequences of aerobic methanotrophs, including *M. album* strain BG8, points to the frequent presence of putative nitrite and nitric oxide reductases, while only three cultivated methanotrophs possess a respiratory nitrate reductase (Stein and Klotz, 2011; Stein et al., 2011; Svenning et al., 2011; Khadem et al., 2012b; Vuilleumier et al., 2012; Kits et al., 2013). It is also unclear whether methanotrophs that lack a respiratory nitrate reductase but possess dissimilatory nitrite and nitric oxide reductases are still capable of denitrification from NO_2_^-^. Moreover, due to the significant divergence of the methanotroph *nirS* from known sequences, it is not known, whether *nirS* is the operational nitrite reductase in the methanotrophs that lack a *nirK* (Wei et al., 2015). While the genome of the nitrate respiring *M. denitrificans* FJG1 encodes both *nirS* and *nirK* nitrite reductases, transcript levels of only *nirK* increased in response to denitrifying conditions (Kits et al., 2015).

The goal of the present study was to test whether a variety of C_1_, C_2_, and inorganic energy sources can directly support denitrification, characterize the environmental factors that regulate NO_2_^-^-dependent N_2_O production in *M. album* strain BG8 and to assess the expression of its putative denitrification inventory.

## Materials and Methods

### Cultivation

*Methylomicrobium album* strain BG8 was cultivated in 100 mL of nitrate mineral salts medium containing 11 mM KNO_3_ (NMS) or 10 mM KNO_3_ plus 1 mM NaNO_2_ (NMS + NO_2_^-^) in 300 mL glass Wheaton bottles topped with butyl rubber septa (Whittenbury et al., 1970). The NMS media was buffered to pH 6.8 using a phosphate buffer (0.26 g/L KH_2_PO_4_, 0.33 g/L Na_2_HPO_4_). The final concentration of copper (CuSO_4_) was 5 μM. Using a 60 mL syringe (BD) and a 0.22 μm filter/needle assembly, CH_4_ (99.998%) was added into the sealed bottles as a sole carbon source. The initial gas-mixing ratio in the headspace was adjusted using O_2_ gas (99.998%, Praxair) to 1.6:1, CH_4_ to O_2_ (or ca. 28% CH_4_, 21% O_2_). The initial pressure in the gas tight bottles was adjusted to ca. 1.3 atm to prevent a vacuum from forming during growth as gas samples and liquid culture samples were withdrawn every 12 h for analysis. Cultures were incubated at 30°C and shaken at 200 rpm. To track growth, the cultures were periodically sampled using a needle fitted syringe (0.5 mL) and cell density was determined by direct count with phase contrast microscopy using a Petroff–Hausser counting chamber. Six biological replicates were grown on separate days and data was collected on each replicate (*n* = 6). Culture purity was assessed by 16s rRNA gene sequencing, phase contrast microscopy, and plating on nutrient agar and TSA with absence of growth indicating no contamination. We assessed purity of the cultures prior to beginning all of the experiments and then assessed it again for each replicate at the conclusion of each experiment.

### Gas Analysis

Concentrations of O_2_, CH_4_, and N_2_O were determined by sampling the headspace of each culture using gas chromatography (GC-TCD, Shimadzu GC8A; outfitted with a molecular sieve 5A and a Hayesep Q column, Alltech). The headspace of each batch culture was sampled with a 250 μL gastight syringe (SGE Analytical Science; 100 μL/injection) at 0 (immediately post inoculation), 6, 12, 16, 20, 24, 30, 36, 42, 48, 60, 72, 96, and 120 h. A total of 200 μL was sampled from each replicate at every time point. We determined the bottles were gastight by leaving a replicate set of bottles uninoculated throughout the experiment and measuring headspace gas concentrations; leakage was <1% over 120 h. Standard curves using pure gases O_2_, CH_4_, and N_2_O (Praxair) were generated and used to calculate the headspace concentrations in the batch cultures.

### Instantaneous Micro-sensor Assays

*Methylomicrobium album* strain BG8 was grown in NMS + NO_2_^-^ medium as described above. At 96 h of growth, when denitrification activity was highly evident, 4 × 10^10^ cells were harvested using a filtration manifold onto 0.2 μm filters (Supor 200, 47 mm, Pall Corporation). The biomass was washed three times with sterile, nitrogen-free mineral salts medium – identical to the mineral salts medium used for cultivation but devoid of NH_4_Cl, KNO_3_, or NaNO_2_. For data presented in **Figures 2** and **4**, the washed biomass was resuspended in the same nitrogen-free medium and transferred to a gastight 10 mL micro-respiration chamber equipped with an OX-MR O_2_ micro-sensor (Unisense) and an N_2_O-500 N_2_O micro-sensor (Unisense). For data presented in **Figure 3**, biomass was resuspended in mineral salts medium amended with 100 μM NaNO_2_. Data was logged using SensorTrace Basic software. CH_4_ gas, 0.001% CH_3_OH (HPLC grade methanol, Fisher Scientific), 0.01% CH_2_O (Methanol free 16% formaldehyde, Life technologies), 10 mM HCO_2_H, C_2_H_6_ gas (99.999%), 0.01% C_2_H_6_O (Methanol free 95% ethyl alcohol, Commercial Alcohols), 200 mM NH_4_Cl, and/or 1 M NO_2_^-^ was injected directly into the chamber through the needle injection port with a gas-tight syringe (SGE Analytical Science). In **Figures 3B–E**, the dissolved O_2_ was decreased to <100 μmol/L (**Figure 3B**) and <25 μmol/L (**Figures 3C–E**), respectively, with additions of CH_4_ (**Figure 3A**), CH_3_OH (**Figure 3B**), CH_2_O (**Figure 3C**), HCO_2_H (**Figure 3D**), C_2_H_6_ (**Figure 3E**), C_2_H_6_O (**Figure 3F**) before data logging was enabled to limit the traces to <100 min and to reduce the number of sampling points. NO_2_^-^ concentration was determined using a colorimetric method (Bollmann et al., 2011). Experiments were performed 3–4 times to demonstrate reproducibility of results and a single representative experiment was selected for presentation.

### RNA Extraction

Total RNA was extracted from ca. 10^9^ *M. album* strain BG8 cells grown in NMS or NMS + NO_2_^-^ medium at 24, 48, and 72 h using the MasterPure RNA purification kit (Epicentre). Briefly, cells were harvested by filtration through a 0.22 μm filter and inactivated with phenol–ethanol stop solution (5% phenol, 95% EtOH). Total nucleic acid was purified according to manufacturer’s instructions with the following modifications: 6 U proteinase K (Qiagen) were added to the cell lysis step and the total precipitated nucleic acid was treated with 30 units of DNase I (Ambion). The total RNA was then column-purified using RNA clean & concentrator (Zymo Research). RNA quality and quantity was assessed using BioAnalyzer (Agilent Technologies) and Qubit (Life Technologies). Residual genomic DNA contamination was assessed by quantitative PCR (qPCR) targeting *norB1* or *nirS* genes (primers listed in **Table 1**). PCR conditions are described below. The total RNA samples were deemed free of genomic DNA if no amplification was detected after 40 cycles of qPCR. High quality RNA (RIN number >9, no gDNA detected) was converted to first strand cDNA using Superscript III reverse transcriptase (Life Technologies), according to manufacturer’s instructions.

**Table 1:** qPCR Primers used in this study.

Gene target	Locus tag^1^	Primer set	Sequence (5′→3′)	qPCR efficiency	Standard curve – R^2^	Reference
*pxmA*	METAL_RS06980	QpxmA-FWD-3	GCTTGTCAGGGCTTACGATTA	97.7% – 101.8%	0.9989	This study
		QpxmA-REV-3	CTTCCAGTCCACCCAGAAATC			
*pmoA*	METAL_RS17425	QpmoA-FWD-7	GTTCAAGCAGTTGTGTGGTATC	95.1% – 97.2%	0.9999	This study
		QpmoA-REV-7	GAATTGTGATGGGAACACGAAG			
*nirS*	METAL_RS10995	QnirS-FWD-1	GTCGACCTGAAGGACGATTT	95.1% – 98.8%	0.9999	This study
		QnirS-REV-1	GTCACGATGCTGTCGTCATA			
*norB1*	METAL_RS03925	QnorB-FWD-2	ACTGGCGGTGCACTATTT	97.2% – 97.4%	0.9998	This study
		QnorB-REV-2	CATCCGGTTGACGTTGAAATC			
*norB2*	METAL_RS13345	QnorB-F-1	CACCATGTACACCCTCATCTG	96.2% – 102.2%	0.9999	This study
		QnorB-R-1	CCAAAGTCTGCGCAAGAAAC	96.1% – 101.8%	0.9999	This study
16S rRNA	METAL_RS04240	341F	CCTACGGGAGGCAGCAG	96.9% – 102.2 %	0.9997	Muyzer et al., 1993
		518R	ATTACCGCGGCTGCTGG			

*^1^The complete genome sequence of *M. album* strain BG8 is deposited in Genbank (http://www.ncbi.nlm.nih.gov/genbank/) under the accession NZ_CM001475 (http://www.ncbi.nlm.nih.gov/genome/?term=methylomicrobium%20album%20Bg8)*.

### Quantitiative PCR

Gene copy standards were created using the genomic DNA of *M. album* strain BG8 using universal and gene-specific primers (**Table 1**). A 10-fold dilution series (10^0^–10^8^ copies/20 μl reaction) of purified amplicons was prepared and used to establish an optimized qPCR condition. Each 20 μl reaction contained 10 μl of 2X qPCR SYBR based master mix (MBSU, University of Alberta), 0.2 μM of forward and reverse primer, 1 μl diluted cDNA, and nuclease-free water. Amplification was performed on a StepOne Plus qPCR system (Applied Biosystems) with an initial activation at 95°C for 3 min and fluorescence emission data collected from 40 cycles of amplification (95°C for 15 s, 60°C for 15 s, and 72°C for 15 s). Target specificity was assessed by melt curve analysis, which ensured that a single peak was obtained. Gene copy number was estimated from cDNA diluted from 10^-3^ to 10^-5^ copies for 16S rRNA and *pmoA* transcript analyses and dilutions from 10^-1^ to 10^-3^ copies for *nirS*, *norB1*, *norB2*, and *pxmA* transcript analyses. The transcript abundance of each functional gene was normalized to that of 16s rRNA to yield a copy number of transcripts per one billion copies of 16s rRNA. Then, to calculate the N-fold change, we divided the transcript abundance (per one billion copies of 16s rRNA) in the NMS + NO_2_^-^ cultures by transcript abundance (per one billion copies of 16s rRNA) in the NMS cultures. Samples were run in triplicate with three dilutions each on at least three biological replicates from cells grown and processed on separate dates. Quantitative PCR efficiencies ranged from 95–102% with *r*^2^-values of at least 0.99 for all assays (**Table 1**).

### Statistics

A Student’s *t*-test (two tailed) was used to calculate the P-level between the control (NMS alone) and experimental (NMS + NO_2_^-^) replicates as indicated for each experiment. Equal variance between the control and experimental groups was determined using a two sample *F* test for variance. The doubling time, O_2_ and CH_4_ consumption, cell density, and total headspace O_2_ and CH_4_ consumed (Supplementary Table S1) all had equal variance between the control and experimental (*F* < *F*_crit_); thus a homoscedastic *t*-test was calculated for the aforementioned comparisons. For qPCR, comparisons between NMS + NO_2_^-^ and NMS alone at 48 h for *pmoA*, *pxmA*, *nirS*, and *norB1*, as well as for *pxmA* and *nirS* at 72 h showed unequal variance (*F* > *F*_crit_); thus a heteroscedastic *t*-test was used to calculate the P-level for these comparisons. The variance between NMS + NO_2_^-^ and NMS alone for all other genes at all other time points was equal (*F* < *F*_crit_).

## Results

### Growth Phenotype of *Methylomicrobium album* Strain BG8 in the Absence or Presence of NO_2_^-^

**FIGURE 1:**
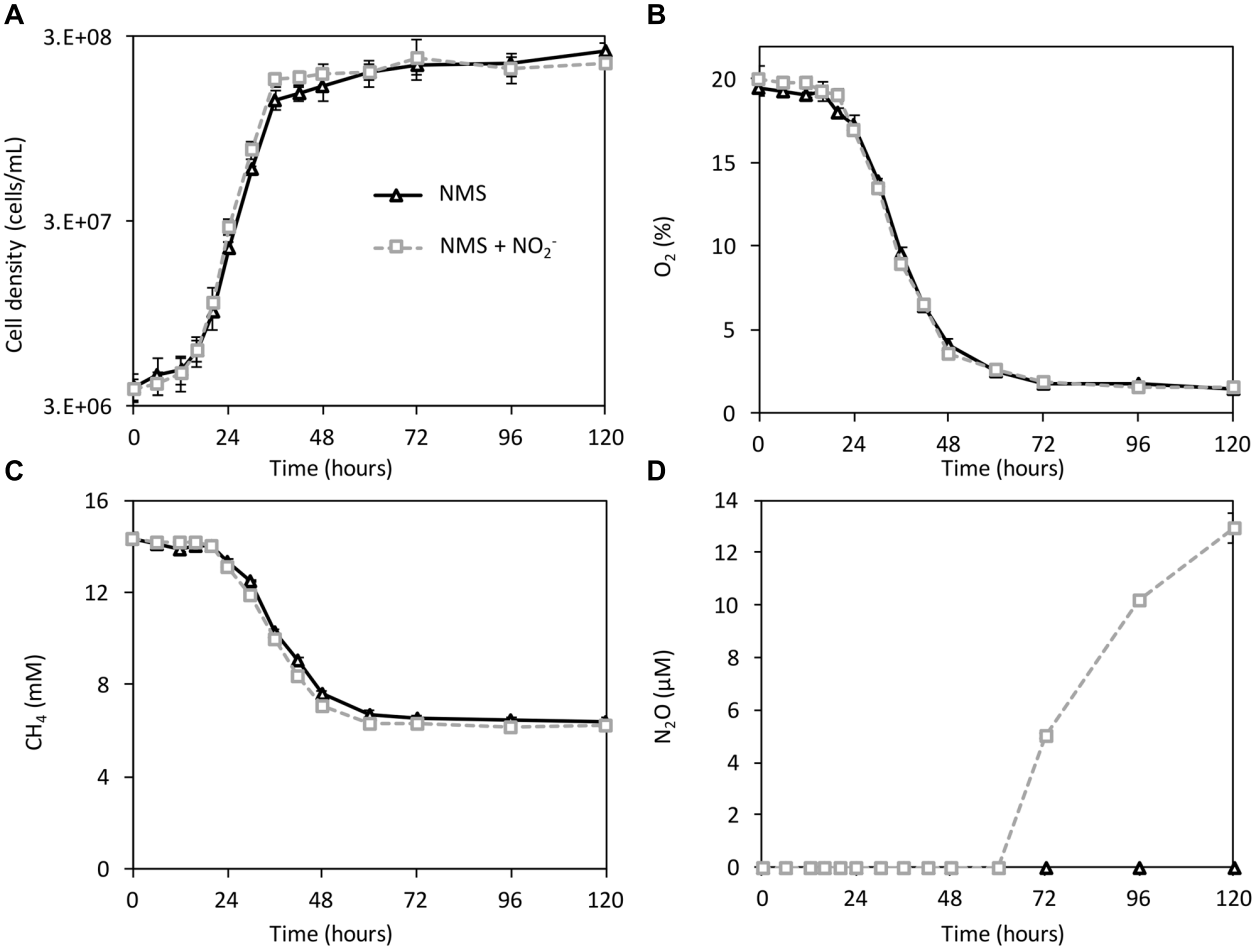
**Growth, CH_4_ and O_2_ consumption, and N_2_O production by *Methylomicrobium album* strain BG8 cultivated on NMS and NMS plus 1 mM NaNO_2_**. *Methylomicrobium album* strain BG8 was cultivated for 5 days in 100 mL of NMS (black triangles) or NMS + 1 mM NO_2_^-^ (gray dashed squares) media in 300 mL closed glass Wheaton bottles sealed with butyl rubber septum caps. The initial headspace gas-mixing ratio of CH_4_ to O_2_ was 1.6:1. Cell density **(A)** was measured using direct count with a Petroff–Hausser counting chamber and headspace gas concentrations of O_2_ **(B)**, CH_4_ **(C)** and N_2_O **(D)** were measured using GC-TCD. All data points represent the mean ± SD for six biological replicates (*n* = 6).

*Methylomicrobium album* strain BG8 was cultivated in NMS or NMS supplemented with NO_2_^-^ over 120 h to determine the effect of NO_2_^-^ on growth, O_2_ and CH_4_ consumption, and N_2_O production (**Figure 1**). The total amount of nitrogen was kept constant to eliminate a difference in N-availability and salt concentration between treatments. All of the cultures were initiated at an oxygen (O_2_) tension of 19.5 ± 0.7% (**Figure 1B**). As observed previously (Nyerges et al., 2010), NO_2_^-^ amendment (1 mM) did not have an inhibitory effect on growth or substrate consumption of *M. album* strain BG8 (**Figures 1A–C** and Supplementary Table S1). The limiting substrate in all treatments was O_2_, as demonstrated by supplementing cultures with additional O_2_ (20 mL) after 48 h of growth and observing a significant increase in optical density in comparison to cultures not receiving additional O_2_ (Supplementary Figure S1). N_2_O production occurred only in the NMS plus NO_2_^-^ cultures (**Figure 1D**). N_2_O production was first apparent in the headspace of NO_2_^-^ amended cultures at 72 h of growth when O_2_ reached ca. 1.8% of the headspace and continued up to the termination of the experiment (120 h) at a rate of 9.3 × 10^-18^ mol N_2_O per cell per hour (**Figure 1D**). After 120 h of growth, the N_2_O yield percentage from the added NO_2_^-^ (100 μmol) was 5.1 ± 0.2% (5.1 ± 0.2 μmol) in the NMS + NO_2_^-^ cultures.

### O_2_ Consumption and N_2_O Production by Resting Cells of *M. album* Strain BG8 with Single or Double Carbon Substrates or Ammonium under Atmospheric and Hypoxic O_2_ Tensions

**FIGURE 2:**
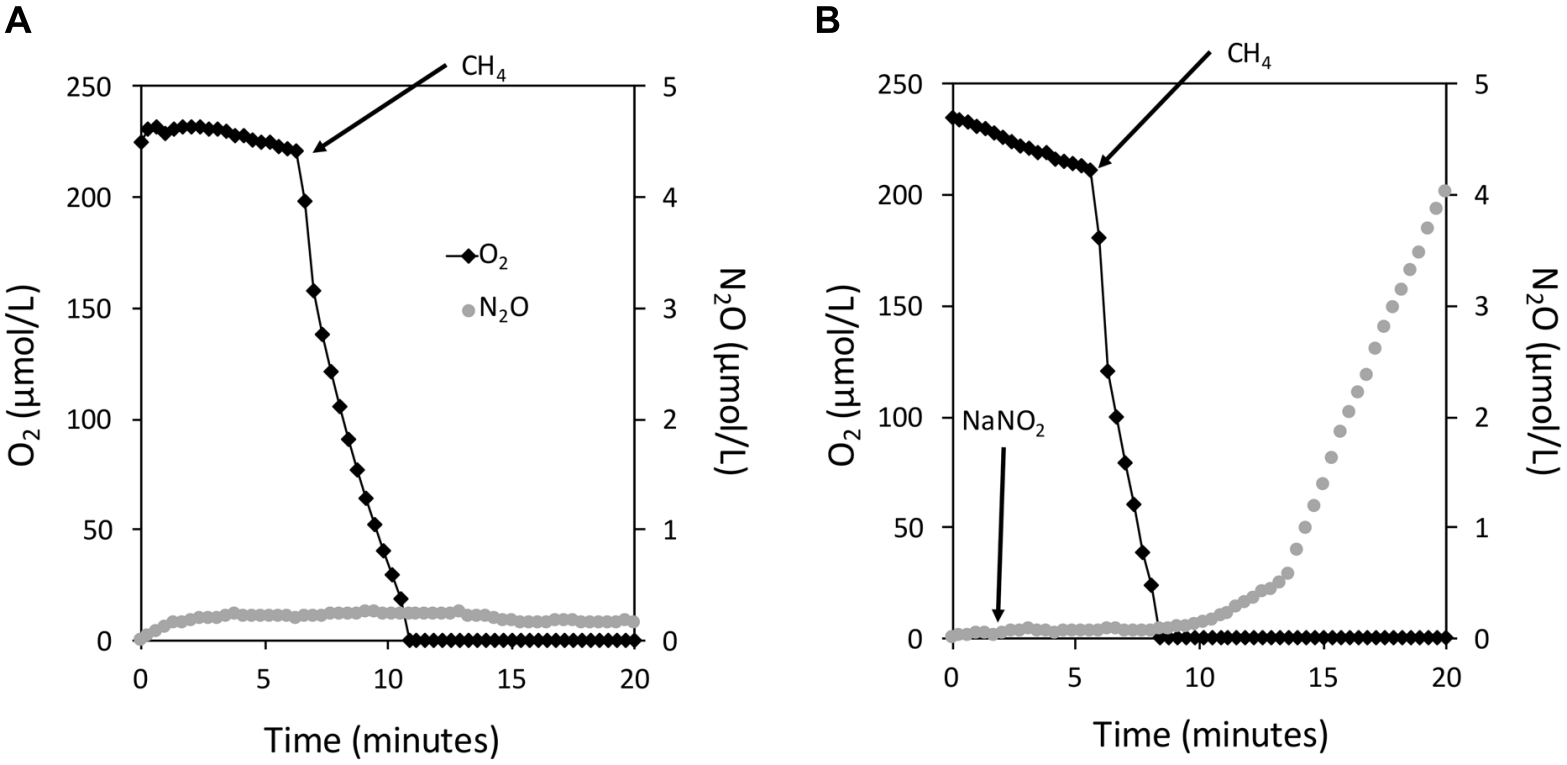
**The instantaneous coupling of CH_4_ oxidation to NO_2_^-^ reduction in *Methylomicrobium album* strain BG8 under hypoxia**. Experiments were performed in a closed 10 mL micro-respiratory chamber outfitted with an O_2_ and N_2_O microsensor and logged with Sensor Trace Basic software. O_2_ (black diamonds) and N_2_O (gray circles) were measured using microsensors. Cells of *M. album* strain BG8 were harvested as described in the materials and methods and resuspended in nitrogen free mineral salts medium. Arrows mark the addition of CH_4_ (∼300 μM) and NaNO_2_ (1 mM) to the micro-respiratory chamber in all panels. There is no measureable denitrification activity in the absence of NO_2_^-^ **(A)**; denitrification activity is dependent on CH_4_ and NO_2_^-^ **(B)**.

To determine which conditions govern N_2_O production in *M. album* strain BG8, we measured instantaneous O_2_ consumption and N_2_O production by *M. album* strain BG8 with CH_4_ as the sole carbon and energy source in a closed 10-mL micro-respiratory (MR) chamber outfitted with O_2_ and N_2_O-detecting microsensors. Introduction of CH_4_ (300 μM) into the chamber led to immediate O_2_ consumption; O_2_ declined to below the detection limit of the sensor (<50 nM O_2_) after ca. 3 min (**Figure 2A**). Addition of NO_2_^-^ to the chamber led to production of N_2_O shortly after O_2_ declined below the detection limit at a rate of 7.9 × 10^-18^ mol cell^-1^ h^-1^ (**Figure 2B**). In the absence of NO_2_^-^, we observed no measureable N_2_O production (**Figure 2A**). Though the O_2_ concentration is <50nM O_2_ when N_2_O production is evident, it is important to note that *M. album* strain BG8 still requires O_2_ for methane oxidation and cannot grow on CH_4_ anaerobically.

**FIGURE 3:**
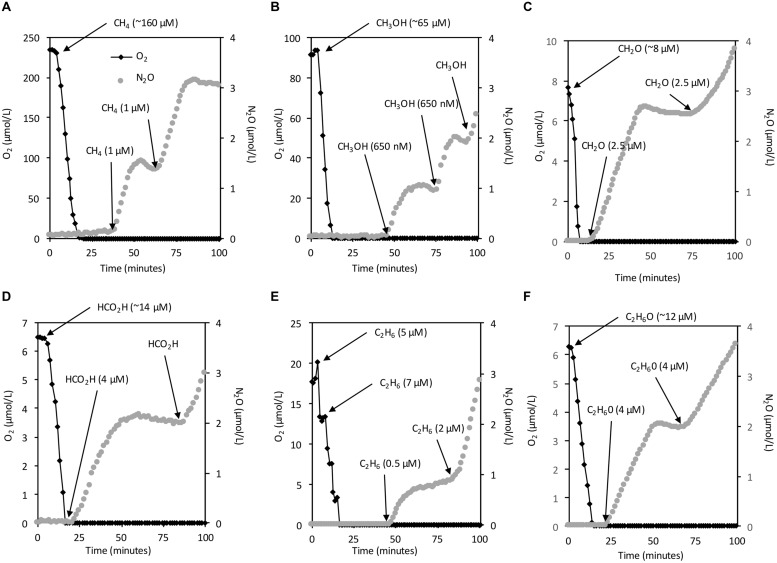
**NO_2_^-^ reduction to N_2_O by *M. album* strain BG8 is dependent on an energy source at <50 nM O_2_**. Experiments were performed in a closed 10 mL micro-respiratory chamber outfitted with an O_2_ and N_2_O microsensor and logged with Sensor Trace Basic software. O_2_ (black diamonds) and N_2_O (gray circles). Cells of *M. album* strain BG8 were harvested as described in the materials and methods and resuspended in mineral salts medium containing 100 μM NO_2_^-^. Arrows mark the addition of either CH_4_ **(A)**, CH_3_OH **(B)**, CH_2_O **(C)**, HCO_2_H **(D)**, C_2_H_6_ **(E)**, C_2_H_6_O **(F)**, in all panels. The right *y*-axis is identical in all panels. However, it should be noted that the left *y*-axis differs in all panels.

Using the same setup described above, we supplemented resting cells in the MR chamber with CH_3_OH, CH_2_O, HCO_2_H, C_2_H_6_, or C_2_H_6_O to experimentally address whether carbon-based reductant sources other than CH_4_ support denitrification in *M. album* strain BG8. Also, to substantiate that the one- and two-carbon sources we tested can all serve as direct electron donors for denitrification by *M. album* strain BG8 under hypoxia, we provided resting cells only enough reductant to consume the dissolved O_2_ (ca. 234 μmol/L) present in the MR chamber sparing no reductant to support denitrification (**Figure 3**). We then measured instantaneous N_2_O production through serial addition of small quantities of CH_4_, CH_3_OH, CH_2_O, HCO_2_H, C_2_H_6_, or C_2_H_6_O to the MR chamber, which contained medium supplemented with NaNO_2_ (100 μM; **Figure 3**). For all six substrates, N_2_O production was stoichiometric with the amount of added substrate (**Figure 3**).

**FIGURE 4:**
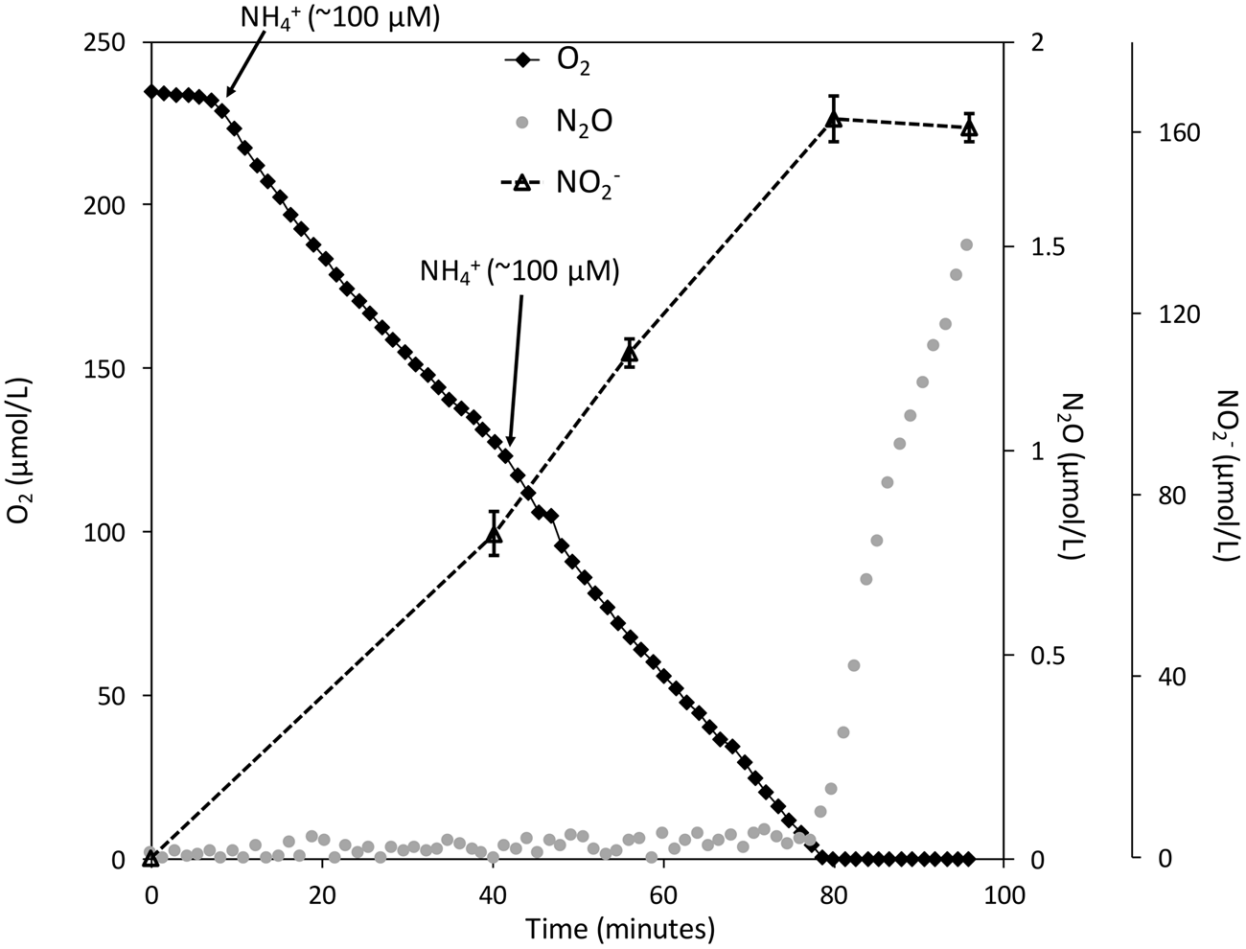
**The coupling of NH_3_ oxidation to NO_2_^-^ reduction in *Methylomicrobium album* strain BG8 under hypoxia**. Experiments were performed in a closed 10 mL micro-respiratory chamber outfitted with an O_2_ and N_2_O microsensor and logged with Sensor Trace Basic software. O_2_ (black diamonds), N_2_O (gray circles), NO_2_^-^ (black dashed triangles). Cells of *M. album* strain BG8 were grown and harvested as described in the materials and methods and resuspended in nitrogen free mineral salts medium. Arrows mark the addition of NH_4_^+^ (100 μM) to the closed micro-respiratory chamber. Traces (O_2_ + N_2_O) are single representatives of reproducible results from cultures grown on different days. NO_2_^-^ was measured using a colorimetric method as described in the Section “Materials and Methods” and data points represent the mean ± SD for three technical replicates.

Many methanotrophs, including *M. album* BG8, can oxidize NH_3_ to NO_2_^-^ due to homologous inventory to ammonia-oxidizing bacteria (Yoshinari, 1985; Bedard and Knowles, 1989; King and Schnell, 1994; Holmes et al., 1995; Poret-Peterson et al., 2008; Campbell et al., 2011; Stein and Klotz, 2011). We aimed to test whether reductant and NO_2_^-^ from NH_3_ oxidation could also drive denitrification by *M. album* strain BG8. Resting cells in the MR chamber consumed the dissolved O_2_ promptly after NH_4_Cl (200 μM) was injected into the chamber (**Figure 4**). After ca. 70 min, the biomass depleted the dissolved O_2_ to <50 nM and NO_2_^-^ concentration reached 163 ± 5 μM. The rate of N_2_O production following O_2_ depletion was 1.2 × 10^-18^ mol cell^-1^ h^-1^.

### Expression of Predicted Denitrification Genes in *M. album* Strain BG8 under Denitrifying Conditions

The genome of *M. album* strain BG8 encodes several genes predicted to be involved in denitrification. The first step in respiratory denitrification is the one-electron reduction of NO_3_^-^ to NO_2_^-^; a reaction performed by one of two membrane-associated dissimilatory nitrate reductase enzymes, neither of which is encoded in the *M. album* strain BG8 genome (Kits et al., 2013). The second step in denitrification, the one-electron reduction of NO_2_^-^ to NO is carried out by one of two non-homologous nitrite reductases, either a copper containing (*nirK*) or a cytochrome cd_1_ containing (*nirS*) nitrite reductase, of which the latter was annotated in the genome (Kits et al., 2013). The genome of *M. album* strain BG8 also contains two copies of a putative cytochrome *c*-dependent nitric oxide reductase (*norB1* and *norB2*, respectively). We also investigated expression of the *pxmA* gene of the *pxmABC* operon that encodes a CuMMO with evolutionarily relatedness to particulate methane monooxygenase (Tavormina et al., 2011). We chose to examine expression of *pxmA* in *M. album* strain BG8 to determine whether this gene responded similarly to that of *M. denitrificans* FJG1; expression of the *pxmABC* operon in *M. denitrificans* FJG1 significantly increased in response to denitrifying conditions (Kits et al., 2015).

**FIGURE 5:**
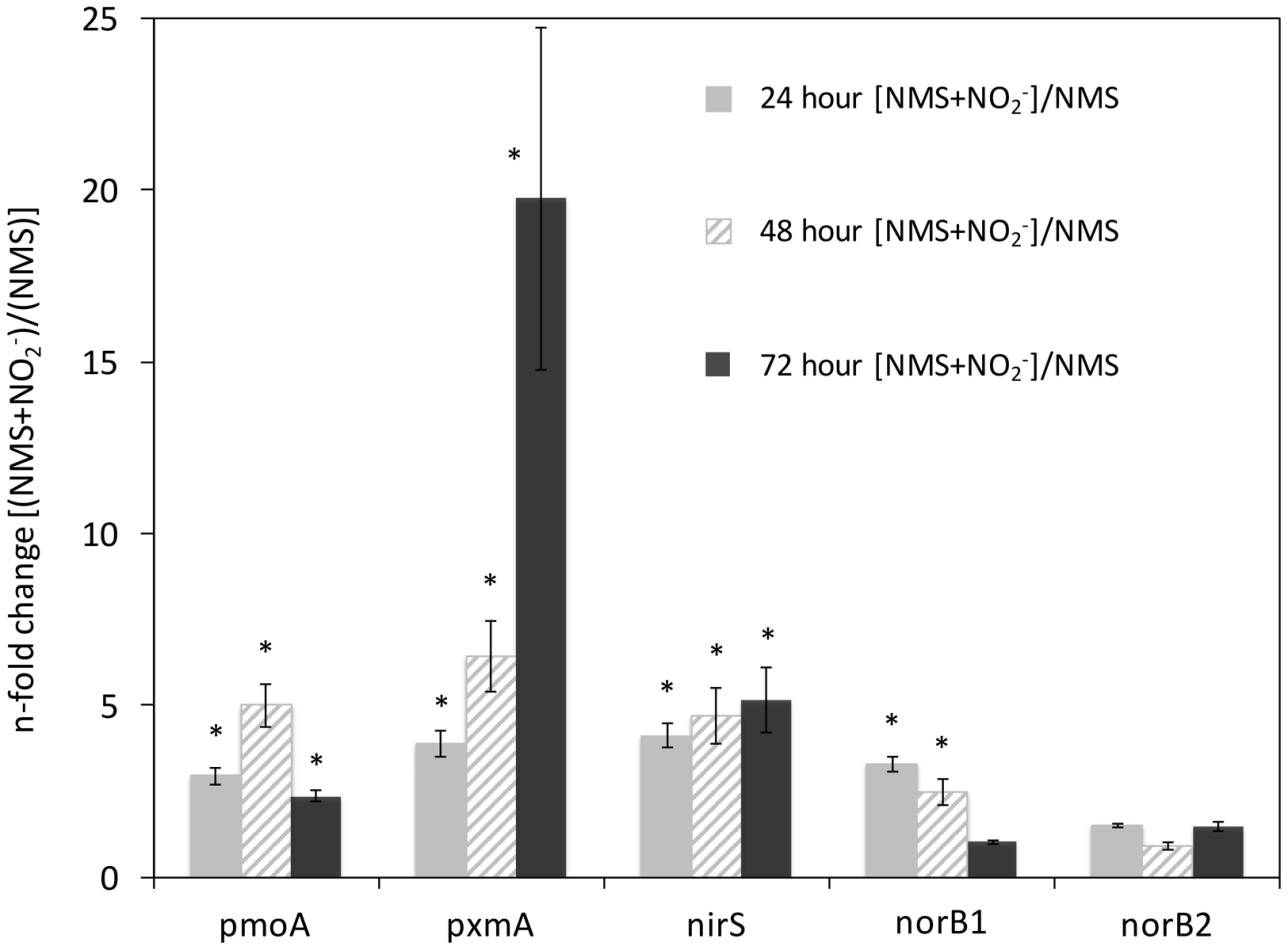
**Expression of *pmoA*, *pxmA*, *nirS*, *norB1*, and *norB2* in *Methylomicrobium album* strain BG8 cultivated in NMS or NMS media amended with 1 mM NaNO_2_**. Total RNA was extracted from *Methylomicrobium album* strain BG8 at 24, 48, and 72 h of growth (see **Figure 1**) from three separate cultures, converted to cDNA, and the abundance of *pmoA*, *pxmA*, *nirS*, *norB1*, and *norB2* transcripts was determined using quantitative PCR. The transcript abundance of each gene of interest was normalized to that of 16s rRNA. The n-fold change in transcript abundance of the NO_2_^-^ amended (1 mM NaNO_2_) NMS cultures relative to the unamended NMS cultures at 24 h of growth (light gray), 48 h of growth (diagonal white/gray), and at 72 h of growth (black). Error bars represent the SD calculated for triplicate qPCR reactions performed on each of the three biological replicates for each treatment. The (^∗^) above the bars designates a statistical significance (*P* < 0.05) as determined by *t*-test between NMS only and NMS + NO_2_^-^ for each time point.

To assess the effect of NO_2_^-^ amendment on gene expression, we used cultures grown in NMS alone as the control. The O_2_ concentration in the headspace of NMS and NMS + NO_2_^-^ cultures after 24 h growth was ca. 17.2 and 16.9%, respectively (**Figure 1B**). The transcript levels of *pmoA, pxmA, nirS*, and *norB1* were significantly higher at the 24 and 48 h time points in the NO_2_^-^ amended cultures when compared to the NMS alone (**Figure 5**). At the 72 h time point, levels of *pmoA* and *nirS* transcript levels remained significantly elevated in the NMS + NO_2_^-^ relative to the NMS only cultures, whereas expression of *norB1* was no longer significantly elevated (**Figure 5**). Most interestingly, the transcript abundance of *pxmA* at 72 h was 19.8-fold higher in NMS + NO_2_^-^ relative to NMS only cultures (**Figure 5**). The second copy of *norB* (*norB2*) was unresponsive (below twofold) to NO_2_^-^ amendment at all time points sampled.

## Discussion

### *Methylomicrobium album* Strain BG8 Produces N_2_O Only as a Function of Hypoxia and NO_2_^-^

Batch cultivation of *M. album* BG8 clearly revealed that both NO_2_^-^ and low O_2_ were required for denitrification, as measured by N_2_O production. Although batch cultures of *M. album* strain BG8 have been shown to produce N_2_O previously in end-point assays (Nyerges et al., 2010), the mechanism and required conditions for denitrification by this strain were not determined until now. N_2_O production by *M. denitrificans* FJG1 was also shown to be dependent on hypoxia (Kits et al., 2015); however, this strain was able to respire NO_3_^-^ in addition to NO_2_^-^ likely due to the presence of a *narGHJI* dissimilatory nitrate reductase that is absent in the genome of *M. album* strain BG8. The genome of *M. album* strain BG8 encodes putative dissimilatory nitrite (*nirS*) and nitric oxide (*norB*) reductases (Kits et al., 2013) like *M. denitrificans* FJG1; hence, it is likely that N_2_O by *M. album* strain BG8 is from the enzymatic reduction of NO_2_^-^ to N_2_O via the intermediate NO.

The correlation between N_2_O production and low O_2_ tension is similar to two other microbial processes, aerobic denitrification in heterotrophic bacteria such as *Paracoccus denitrificans* and nitrifier denitrification in ammonia-oxidizing bacteria (Richardson et al., 2001; Kozlowski et al., 2014). Aerobic denitrification in chemoorganoheterotrophs and nitrifier-denitrification in ammonia-oxidizing bacteria is a tactic used to maximize respiration during O_2_ limitation or to expend surplus reductant (Richardson et al., 2001; Stein, 2011). Utilization of NO_2_^-^ in combination with or instead of O_2_ in the respiratory chain of *M. album* strain BG8 would reduce the overall cellular O_2_ demand, thus conserving O_2_ for additional CH_4_ oxidation. Thus, it is possible that *M. album* strain BG8 uses NO_2_^-^ as a terminal electron acceptor under O_2_ limitation to maximize total respiration. The N_2_O yield percentage from NO_2_^-^ by *M. album* strain BG8 (5.1 ± 0.2%) is similar to that of *Nitrosomonas europaea* ATCC 19718 (ca. 4.8%) and one order of magnitude higher than that of *Nitrosospira multiformis* ATCC 25196 (0.27 ± 0.05%; Kozlowski et al., 2014; Stieglmeier et al., 2014).

### Denitrification by *M. album* Strain BG8 is Enzymatically Supported by Diverse Reductant Sources

Resting cells of *M. album* strain BG8 reduced NO_2_^-^ to N_2_O at the expense of any of four tested C_1_ substrates (CH_4_, CH_3_OH, CH_2_O, HCO_2_H), the two C_2_ substrates (C_2_H_6_, C_2_H_6_O), and NH_4_Cl. These data show that intermediates of the methanotrophic pathway and co-substrates of pMMO, MDH, and likely hydroxylamine dehydrogenase support respiratory denitrification. These results agree with previous work on the methanotroph *Methylocystis* sp. strain SC2, which couples CH_3_OH oxidation to denitrification under anoxia (Dam et al., 2013). Remarkably, both C_2_ compounds we tested – C_2_H_6_ and C_2_H_6_O – supported denitrification. The ability of C_2_ compounds to support denitrification in methanotrophs may have environmental significance as natural gas consists of ∼1.8–5.1% (vol%) C_2_H_6_ (Demirbas, 2010). Further, C_2_H_6_O is a significant product of fermentation by primary fermenters during anoxic decomposition of organic compounds (Reith et al., 2002). The results also demonstrate that electrons derived from the oxidation of NH_3_ to NO_2_^-^ were effectively utilized by nitrite and nitric oxide reductases in *M. album* strain BG8, which represents yet another pathway for methanotrophic N_2_O production that is not directly dependent on single-carbon metabolism, provided that the methane monooxygenase can access endogenous reductant (Dalton, 1977; King and Schnell, 1994; Stein and Klotz, 2011).

Instantaneous O_2_ consumption and N_2_O production measurements (**Figures 2–4**) provide strong support that catabolism of C_1_ – C_2_ substrates and ammonia is directly coupled to NO_2_^-^ reduction under hypoxia in *M. album* strain BG8. Some aerobic methanotrophs ferment CH_4_ and excrete organic compounds such as citrate, acetate, succinate, and lactate (Kalyuzhnaya et al., 2013). Some studies also suggest that methanotrophs only support denitrification within CH_4_-fed consortia by supplying these excreted organics to denitrifying bacteria, since methanotrophs were thought incapable of denitrification by themselves (Costa et al., 2000; Knowles, 2005; Liu et al., 2014). Although *M. album* strain BG8 may excrete organic compounds under hypoxia when provided with CH_4_, the ability of CH_3_OH, CH_2_O, HCO_2_H, C_2_H_6_, C_2_H_6_O, or NH_3_ oxidation to support denitrification unequivocally demonstrates the linkage between methanotroph-specific enzymology and denitrifying activity within a single organism.

### Transcription of Predicted Denitrification Genes, *nirS* and *norB1*, Increased in Response to NO_2_^-^ but not Hypoxia

The expression of a *nirS* homolog in an aerobic methanotroph has been investigated so far only in the NO_3_^-^ respiring *M. denitrificans* FJG1 (Kits et al., 2015). Interestingly, the genome of *M. denitrificans* FJG1 encodes both the copper-containing (*nirK*) and cytochrome cd_1_ containing (*nirS*) nitrite reductases and only the steady state mRNA levels of *nirK* increased in this strain in response to simultaneous O_2_ limitation and NO_3_^-^ availability (Kits et al., 2015). In the case of *M. album* strain BG8, which only possesses a *nirS* homolog, we showed that the abundance of this *nirS* transcript responded positively to NO_2_^-^ treatment but not to O_2_ limitation. This suggests that NO_2_^-^ availability alone elicits the expression of *nirS*, even though hypoxia was required for NO_2_^-^ reduction to occur.

The cytochrome *c* dependent nitric oxide reductase (*norB*) is widely found in the genomes of aerobic methanotrophs (Stein and Klotz, 2011). This may in part be due to the need to detoxify NO that is produced during aerobic ammonia oxidation by reducing it to N_2_O (Sutka et al., 2003). The expression of *norB* in *Methylococcus capsulatus* strain Bath increased 4.8-fold after treatment with 0.5 mM sodium nitroprusside, a NO releasing compound (Campbell et al., 2011). It is possible that the NorB protein is involved in detoxification of NO during NH_3_ oxidation in *M. capsulatus* strain Bath, since the genome lacks a dissimilatory nitrite reductase. More recently, it was demonstrated in *M. fumariolicum* strain SolV that transcription of *norB* was upregulated during O_2_ limitation during chemostat growth (Khadem et al., 2012a); however, it is unknown whether *M. fumariolicum* strain SolV can consume NO_2_^-^ or NO. The transcription of *norB* in *M. denitrificans* FJG1 increased 2.8-fold in response to NO_3_^-^ and hypoxia (Kits et al., 2015). While the genome of *M. album* strain BG8 encodes two copies of the *norB* gene, only one copy (*norB1*) is followed by *norC* – the essential cytochrome *c*-containing subunit (Mesa et al., 2002). Although some organisms like *Cupriavidus necator* possess two independent functional nitric oxide reductases (Cramm et al., 1997), the present work illustrates that expression of only *norB1* in *M. album* strain BG8 is responsive to NO_2_^-^ treatment. Although the function of NorB may differ between *M. album* strain BG8 and *M. capsulatus* strain Bath, both bacteria show a similar transcriptional response of *norB* genes to NO_2_^-^ (Campbell et al., 2011).

### Transcript Abundance of *pxmA* Significantly Increased in Response to both NO_2_^-^ and Hypoxia

Genomes of some aerobic methanotrophs belonging to the phylum *Gammaproteobacteria* have been shown to encode a sequence divergent CuMMO protein complex, pXMO (Tavormina et al., 2011). The function and substrate of the putative pXMO protein encoded by the *pxm* operon remains unknown. Previous studies on the *pxm* operon have shown that it is expressed at low levels during growth in *Methylomonas* sp. strain LW13 as well as in freshwater peat bog and creek sediment (Tavormina et al., 2011). Metagenomic sequencing of the SIP-labeled active community in an oilsands tailings pond revealed that *pxmA* sequences were present in the active methanotroph community (Saidi-Mehrabad et al., 2013). Analysis of the transcriptome of *M. denitrificans* FJG1 revealed that steady state mRNA levels of the *pxmABC* operon increased ∼10-fold in response to denitrifying conditions (Kits et al., 2015).

We now demonstrate that expression of *pxmA* in *M. album* strain BG8 is significantly increased in response to both NO_2_^-^ and hypoxia. We did not observe any increase in the expression of *pxmA* in O_2_ limited NMS-only cultures where denitrification was not occurring, suggesting that hypoxia alone is not sufficient to illicit an increase in the steady state mRNA levels. This study adds further support to the observation that expression of *pxmA* is responsive to denitrifying conditions. However, it must be noted that at 72 h in the NO_2_^-^ amended media, absolute transcript abundance of *pxmA* (1 × 10^3^ copies *pxmA*/1 × 10^9^ copies 16s rRNA) was three orders of magnitude lower than absolute transcript abundance of *pmoA* (1 × 10^6^ copies *pxmA*/1 × 10^9^ copies 16s rRNA).

## Conclusion

**FIGURE 6:**
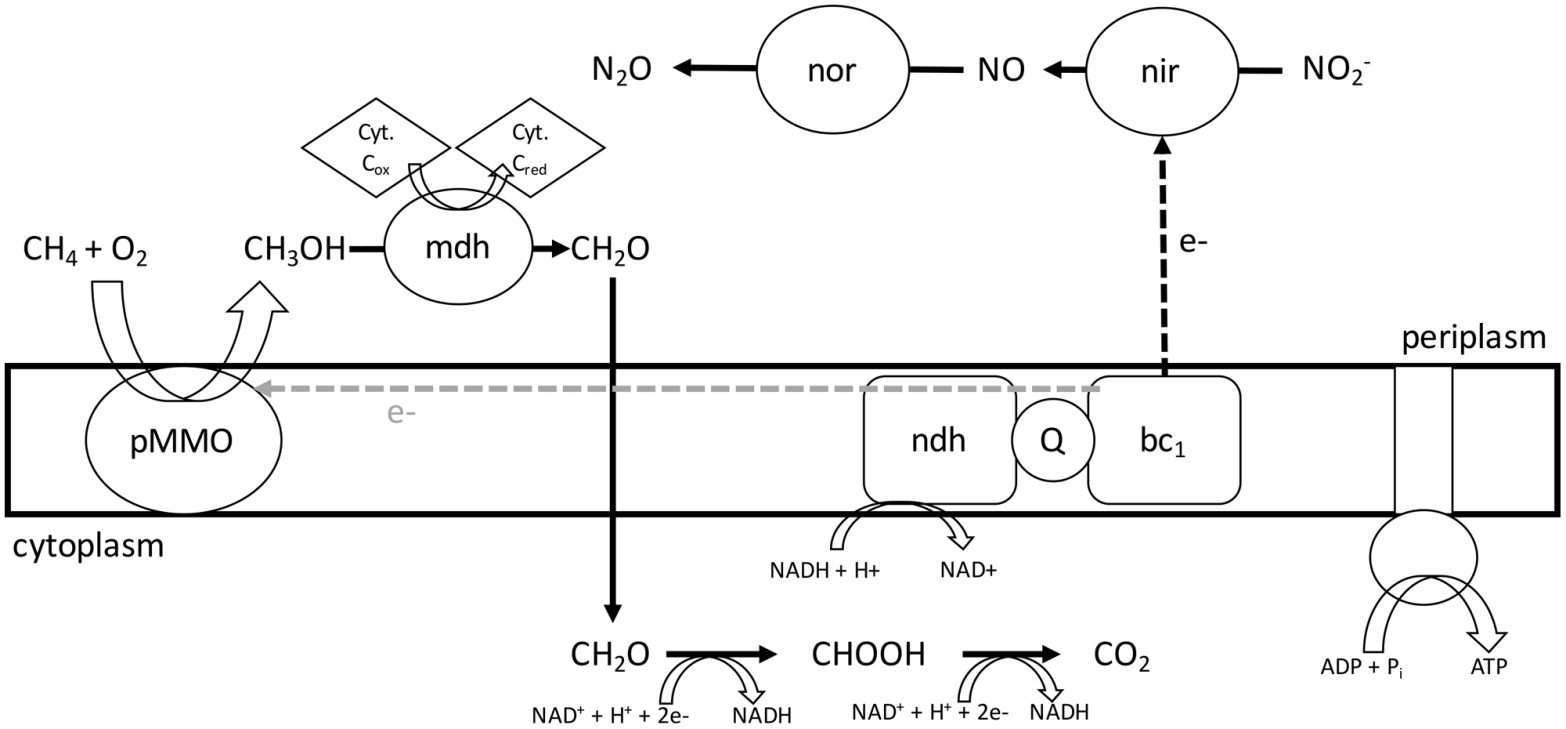
**Proposed model for NO_2_^-^ respiration and central metabolism in *Methylomicrobium album* strain BG8**. During hypoxia, *M. album* strain BG8 utilizes electrons from aerobic CH_4_ oxidation to respire NO_2_^-^. Abbreviations: pMMO, particulate methane monooxygenase; mdh, methanol dehydrogenase; Cyt, cytochrome; nor, nitric oxide reductase; nir, nitrite reductase; ndh, NAD(P)H dehydrogenase complex; Q, coenzyme Q; bc_1_, cytochrome bc_1_ complex.

The present study demonstrates that an aerobic methanotroph – *M. album* strain BG8 – couples the oxidation of C_1_ (CH_4_, CH_3_OH, CH_2_O, HCO_2_H), C_2_ (C_2_H_6_, C_2_H_6_O), and inorganic (NH_3_) substrates to NO_2_^-^ reduction under O_2_ limitation resulting in release of the potent greenhouse gas N_2_O. The ability to couple C_1_, C_2_, and inorganic energy sources to O_2_ respiration and denitrification gives *M. album* strain BG8 considerable metabolic flexibility. We propose a model for methane driven denitrification in *M. album* strain BG8 (**Figure 6**). This discovery has implications for the environmental role of methanotrophic bacteria in the global nitrogen cycle in both N_2_O emissions and N-loss. Comparing the genome and physiology of the NO_2_^-^ respiring *M. album* strain BG8 to NO_3_^-^ respiring *M. denitrificans* FJG1 suggests that the inability of *M. album* strain BG8 to reduce NO_3_^-^ to N_2_O is likely due to the absence of a dissimilatory nitrate reductase in the genome, but that expression of predicted denitrification genes, *nirS* and *norB1*, enable this aerobic methanotroph to respire NO_2_^-^.

## Conflict of Interest Statement

The authors declare that the research was conducted in the absence of any commercial or financial relationships that could be construed as a potential conflict of interest.
