# Supplementary material for: Diverse electron sources support denitrification under hypoxia in the obligate methanotroph Methylomicrobium album strain BG8
Source: Front Microbiol. 2015 Oct 6;6:1072. doi: 10.3389/fmicb.2015.01072 (PMC4594100; doi:10.3389/fmicb.2015.01072)
Supplement: Supplementary file 1 [file Table_1.DOCX]

|  | Table S1: Growth, O_2_ and CH_4_ consumption by pure cultures of *M. album* strain BG8 grown in NMS or NMS + NO_2_^-^ media | | | | | | | | | | |
| --- | --- | --- | --- | --- | --- | --- | --- | --- | --- | --- | --- |
|  | Doubling time (h)^a^ |  | O_2_ consumption (mmol L ^-1^ h^-1^ cell^-1^)^a^ |  | CH_4_ consumption (mmol L ^-1^ h^-1^ cell^-1^)^a^ |  | Final  cell density^b^ |  | Total headspace O_2_ consumed (mM)^c^ |  | Total headspace CH_4_ consumed (mM)^c^ |
| NMS | 4.44 |  | 1.31E-11 |  | 1.45E-11 |  | 2.52E8/mL |  | 8.44 |  | 7.97 |
| NMS + NO_2_^-^ | **4.11^*^** |  | 1.37E-11 |  | 1.37E-11 |  | 2.16E8/mL |  | 8.61 |  | 8.03 |
|  | | | | | | | | | | | |
| ^a^ Cultures of *M. album* strain BG8 were cultivated in NMS media (control) or NMS + 1 mM NO_2_^-^ (experimental) as described in the materials and methods. Linear rates for doubling time were calculated during exponential growth phase (16h – 36h). Linear O_2_ and CH_4_ consumption rates were calculated over the 20 – 48h time interval for both the control and experimental treatments.  ^b^ The maximum cell density of the control and experimental cultures in stationary phase was calculated after 120 h growth as described in the materials and methods.  ^c^ Total O_2_ and CH_4_ consumption was calculated by subtracting the final O_2_ and CH_4_ headspace concentrations (120 h) from those at the beginning of the experiment (0 h) for both the control and experimental cultures.  * Indicates a significant difference (P<0.05) between cultures grown in NMS alone and NMS plus 1 mM NO_2_^-^ . A T-Test was used to determine the P-value when comparing the two treatments (n = 6 for six independent cultures grown on different days). | | | | | | | | | | | |
