# Supplementary figures and images for: Diverse electron sources support denitrification under hypoxia in the obligate methanotroph Methylomicrobium album strain BG8
Source: Front Microbiol. 2015 Oct 6;6:1072. doi: 10.3389/fmicb.2015.01072 (PMC4594100; doi:10.3389/fmicb.2015.01072)

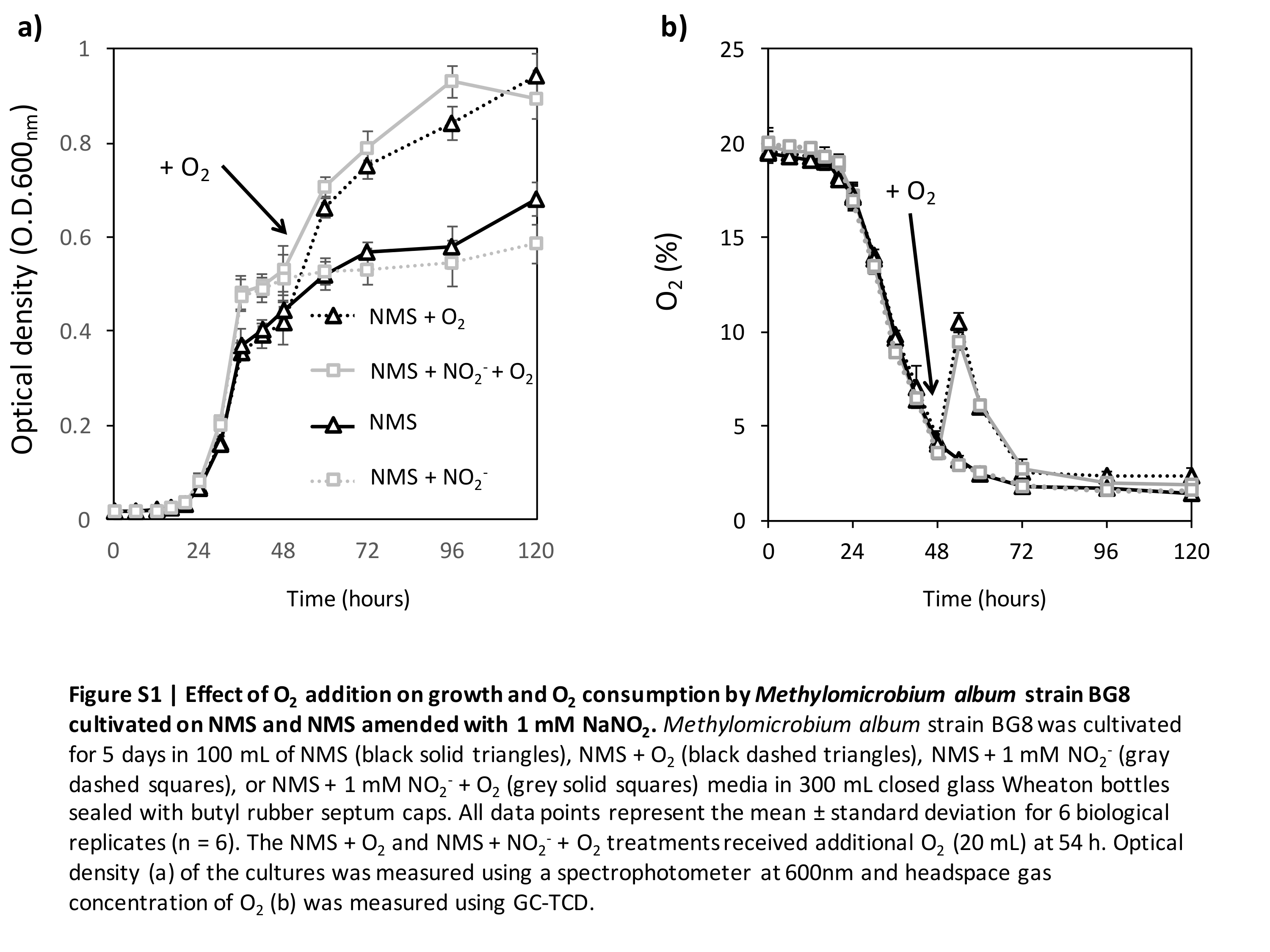

Supplement: Supplementary file 2 [file Image_1.TIF]
